# Pseudocirrhosis Due to Desmoplastic Response to Chemotherapy in Breast Cancer Liver Metastases

**DOI:** 10.7759/cureus.25321

**Published:** 2022-05-25

**Authors:** Kazuhide Takata, Ai Mogi, Ryo Yamauchi, Satoshi Shakado, Fumihito Hirai

**Affiliations:** 1 Department of Gastroenterology and Medicine, Fukuoka University Faculty of Medicine, Fukuoka, JPN; 2 Division of Medical Oncology, Hematology and Infectious Disease, Department of Internal Medicine, Fukuoka University Faculty of Medicine, Fukuoka, JPN

**Keywords:** pseudocirrhosis, portal hypertension, metastatic breast cancer, liver biopsy, fib-4 index

## Abstract

Pseudocirrhosis can result in cirrhosis-like symptoms of portal hypertension and is observed mostly in patients with breast cancer; however, its cause is unclear. Herein, we report a case of pseudocirrhosis in a 76-year-old woman with metastatic breast cancer. The patient developed irregular contours of the liver, resembling cirrhosis, and esophageal varices during chemotherapy for breast cancer with liver metastases. Although intrahepatic metastasis was absent on imaging, a liver biopsy showed cancer cell infiltration consistent with the fibrotic area, which was similar to fibrosis seen in liver cirrhosis. Endoscopic ligation was performed for the varices; however, the patient’s worsening liver function made it difficult to continue chemotherapy. Clinicians should be alert to the possibility of pseudocirrhosis developing in patients with metastatic breast cancer undergoing chemotherapy. Since pseudocirrhosis is a life-threatening complication, non-invasive markers for early diagnosis are needed.

## Introduction

“Pseudocirrhosis” is a radiological term that refers to a condition wherein the hepatic contour shows changes mimicking cirrhosis on imaging. This condition can result in cirrhosis-like symptoms of portal hypertension, including esophageal varices, ascites, and hepatocellular failure [1, 2]. Pseudocirrhosis can occur in a variety of carcinomas, including gastrointestinal cancers and lung adenocarcinomas, but is mostly observed in breast cancer patients. Additionally, the mechanisms associated with the development and progression of pseudocirrhosis remain unknown. Herein, we present a case of pseudocirrhosis in a patient with metastatic breast cancer who underwent chemotherapy.

## Case presentation

**Figure 1:**
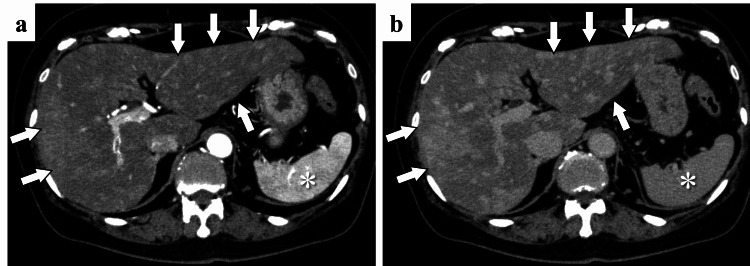
Abdominal enhanced CT performed 18 months prior to the visit to our department (a, b) Early-phase and late-phase of abdominal enhanced CT showing diffusely hypervascular liver metastases (arrows), while there was no evidence of cirrhosis or splenomegaly (*).

A 76-year-old woman was referred to our gastroenterology outpatient clinic for changes in hepatic contour mimicking cirrhosis on abdominal computed tomography (CT) images. Nine years earlier, the patient had been diagnosed with stage-IIB invasive lobular cancer in the left breast (estrogen receptor 0%, progesterone receptor 0%, and human epidermal growth factor-2 negative). She received neoadjuvant chemotherapy with four cycles of nab-paclitaxel and four cycles of 5-fluorouracil, epirubicin, and cyclophosphamide, and underwent a total mastectomy of the left breast with axillary clearance. She received an additional 60 Gy of radiation therapy; however, 17 months post-surgery, bone metastases were observed. She received denosumab, tegafur/gimeracil/oteracil, and eribulin. Five years and seven months after surgery, liver metastasis developed and she received carboplatin (AUC5) and later, irinotecan. Seven years and three months after surgery (18 months before referral to our outpatient clinic), she was started on paclitaxel plus bevacizumab due to increased liver metastasis. At this time, a CT scan showed diffusely hypervascular liver metastases, but no cirrhosis-like changes. Further, fatty liver, which was not present before, was observed (Figure 1a, 1b).

Subsequent germline BRCA mutation tests, and PD-L1 tests performed to evaluate suitability for the administration of immune checkpoint inhibitors were negative. She had no history of alcohol intake.

**Figure 2:**
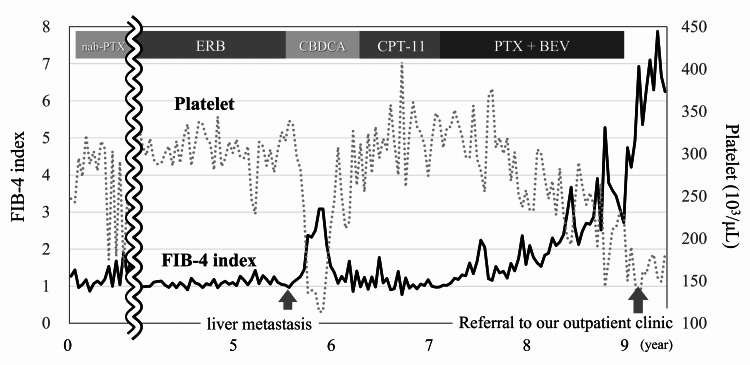
Time course of fibrosis-4 (FIB-4) index levels before referral to our gastroenterology outpatient clinic. nab-PTX, nanoparticle albumin-bound paclitaxel; ERB, eribulin; CBDCA, carboplatin; CPT-11, irinotecan; PTX, paclitaxel; BEV, bevacizumab

The patient was asymptomatic, and her physical examination did not reveal any abnormality. Laboratory tests showed elevated serum liver enzyme levels and fibrosis markers. The fibrosis-4 (FIB-4) index, which is a non-invasive method of assessing liver fibrosis (calculated based on laboratory values and age), increased over time and had a score of 4.33 at the time of referral to our gastroenterology outpatient clinic (Figure 2).

Some tumor markers were elevated (cancer antigen, 15-3 >300 U/mL; carcinoembryonic antigen, 347.7 ng/mL; and carbohydrate antigen, 19-9, 46 U/mL) (Table 1).

**Table 1:** Laboratory data on the first visit. PT, prothrombin time; PT-INR, prothrombin time-international normalized ratio; AST, aspartate aminotransferase; ALT, alanine aminotransferase; ALP, alkaline phosphatase; GGT, gamma-glutamyl transferase; BUN, blood urea nitrogen; eGFR, estimated glomerular ﬁltration rate; M2BPGi, Mac-2 binding protein glycosylation isomer; FIB-4 index, fibrosis-4 index; IgG, Immunoglobulin G; IgM, Immunoglobulin M; ANA, antinuclear antibody; AMA, antimitochondrial antibody; CEA, carcinoembryonic antigen; CA19-9, carbohydrate antigen 19-9; CA15-3, carbohydrate antigen 15-3; DCP, des-c-carboxy prothrombin; sIL-2R, serum interleukin 2 receptor; HCV, hepatitis C virus; HBs, hepatitis B surface; HBc, hepatitis B core.

Hematology		
White blood cell	10300	/μL
Red blood cell	2.87	10^6^/μL
Hemoglobin	8.7	g/dL
Platelets	190	10^3^/μL
Coagulation		
PT	77	%
PT-INR	1.14	
Biochemistry		
Albumin	4.0	g/dL
Total bilirubin	1.8	mg/dL
AST	64	U/L
ALT	35	U/L
ALP	91	U/L
GGT	86	U/L
BUN	19	mg/dL
Creatinine	0.57	mg/dL
eGFR	77.1	mL /min/1.73m²
Glucose	154	mg/dL
HbA1c	5.7	%
M2BPGi	(+) 2.51	COI
Hyaluronic acid	433.9	ng/mL
Type IV collagen 7S	31.7	ng/mL
FIB-4 index	3.55	
IgG	1281	mg/dL
IgM	181	mg/dL
ANA	< 40	Dil
AMA (M2)	(-) < 1.5	Index
Tumor Makers		
CEA	347.7	ng/mL
CA19-9	46	U/mL
CA15-3	> 300	U/mL
Alpha-fetoprotein	2.3	ng/mL
DCP	52	mAU/mL
sIL-2R	522	U/mL
Infectious Makers		
Anti-HCV	(-)	
HBsAg	(-)	
Anti-HBs	(-)	
Anti-HBc	(-)	

**Figure 3:**
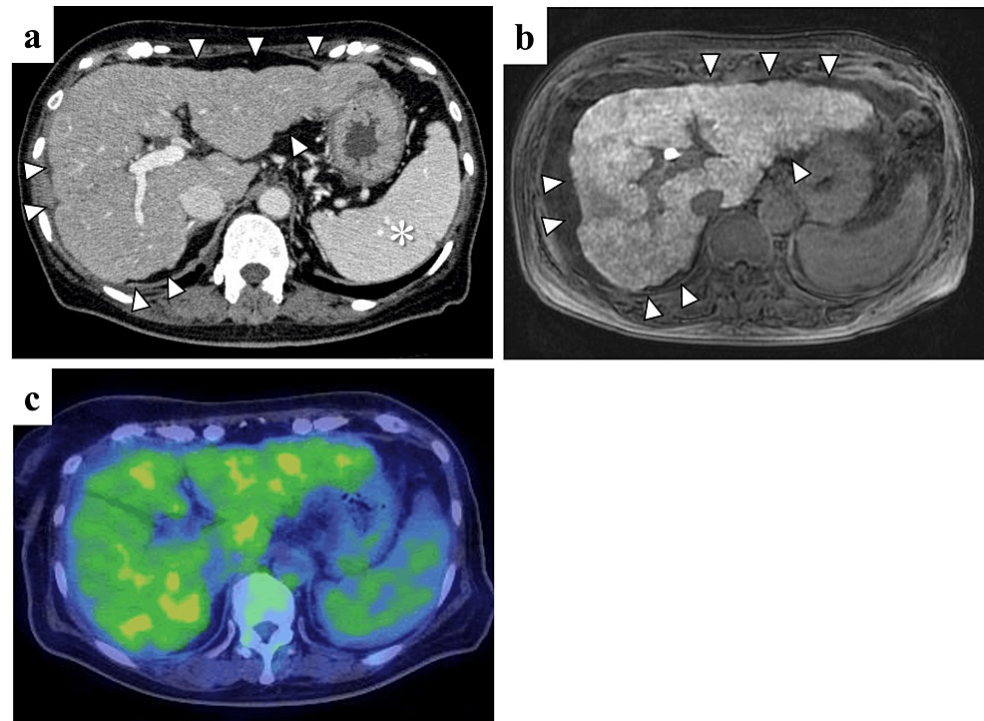
Abdominal images at the time of referral (a, b) Repeated CT and the hepatobiliary phase of gadolinium-ethoxybenzyl-diethylenetriamine-pentaacetic acid-enhanced magnetic resonance imaging showed irregularity of the liver surface (arrowheads), ascites, and splenomegaly (*); however, there were no findings of hepatic metastases. (c) 18F-fluorodeoxyglucose-labeled positron emission tomography-CT scan showed no focal areas of increased uptake to suggest malignancy.

Three-phase dynamic contrast-enhanced CT and gadolinium-ethoxybenzyl-diethylenetriamine-pentaacetic acid (Gd-EOB-DTPA)-enhanced magnetic resonance imaging (MRI) showed cirrhosis-like irregularities, ascites, and splenomegaly that were not present 18 months earlier, but the diffuse liver lesions suggestive of metastasis that were previously present disappeared. Portal vein invasion or thrombus was still absent. In addition, there were no findings suggestive of pancreatic or advanced colorectal cancer. MR elastography revealed an extremely stiff liver (4.2 ± 0.3 kPa; normal <2.5 kPa). On 18F-fluorodeoxyglucose-labeled positron emission tomography-CT (18F-FDG PET-CT), there were no focal areas of increased uptake suggestive of malignancy (Figure 3a-3c).

**Figure 4:**
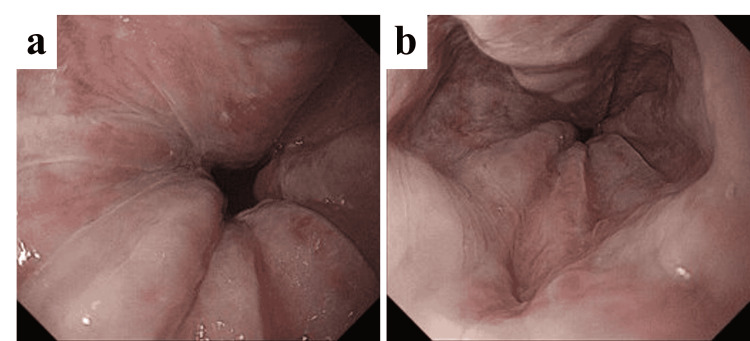
Esophagogastroduodenoscopy images (a, b) Esophagogastroduodenoscopy revealing esophageal varices, classified as Li, F2, Cw, RC0, according to the Japanese Research Society for Portal Hypertension classification.

Esophagogastroduodenoscopy revealed esophageal varices, classified as Li, F2, Cw, RC0, according to the Japanese Research Society for Portal Hypertension classification, with risk of bleeding (Figure 4a, 4b).

**Figure 5:**
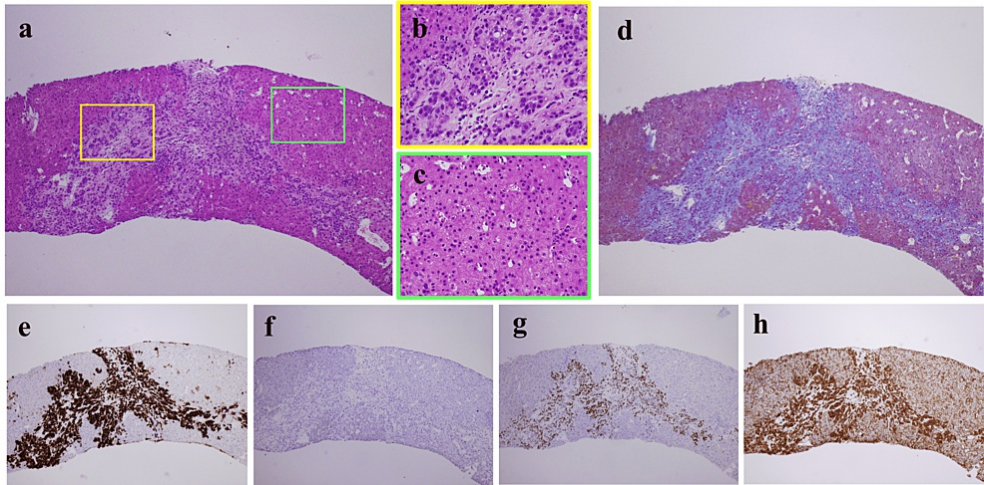
Histological findings from the liver biopsy specimens. (a-d) The histological examination of a liver biopsy specimen shows an island of residual hepatic parenchyma and the surrounding fibrosis that has spread. The fibrosis area is composed of invasive tumor cells. No obvious abnormalities in non-tumor areas. (e-h) Immunohistochemical staining showed that the site of tumor infiltration was positive for CK7, GATA-3, p120-catenin, and negative for CK20. (a, b, c. magnification: ×40 and ×200, hematoxylin and eosin staining; d, magnification: ×40, Masson’s trichrome staining; e, magnification: ×40, CK7; f, magnification: ×40, CK20; g, magnification: ×40, GATA-3; h, magnification: ×40, p120-catenin)

Histological examination of a percutaneous liver biopsy specimen revealed diffuse cancer cell infiltration. Masson’s trichrome staining revealed that these cancer cells were located alongside the fibrotic area that showed findings similar to the fibrosis seen in liver cirrhosis, while immunohistochemical staining revealed that the tumor cells were positive for CK7, GATA-3, and p120-catenin and negative for CK20 (Figure 5a-5h).

Thus, we diagnosed the patient with pseudocirrhosis coupled with esophageal varices due to diffuse metastatic breast cancer.

The patient underwent endoscopic variceal ligation to prevent variceal rupture but was unable to continue chemotherapy for breast cancer due to the rapid worsening of her liver function, and died several weeks later.

## Discussion

Pseudocirrhosis is a life-threatening condition that presents with rapidly progressive cirrhosis-like imaging changes during chemotherapy in patients with breast cancer along with liver metastases [3]. Our patient developed esophageal varices and cirrhosis-like imaging changes during the 18 months of treatment for liver metastases. Involvement of fatty liver or chemotherapy-induced sinusoidal obstruction syndrome (SOS) was ruled out based on histologic findings and on the absence of fatty liver-causing agents such as tamoxifen or SOS-causing agents such as oxaliplatin. Histological examination of the liver biopsy specimen showed cancer cell infiltration consistent with the fibrotic area, and the patient was therefore diagnosed with pseudocirrhosis. Pseudocirrhosis is common, and Oliai et al. reported that the incidence of pseudocirrhosis in breast cancer patients treated with chemotherapy was 20%, all of whom had liver metastases, and the incidence of pseudocirrhosis in cases with liver metastases was 55% [4]. The most common histological type of breast cancer that presented with pseudocirrhosis was invasive ductal carcinoma, followed by invasive lobular carcinoma, which was observed in our case [3,4].

Despite the importance of pseudocirrhosis in determining the prognosis of breast cancer patients, the mechanism underlying the development of pseudocirrhosis remains unclear. Based on previous studies, the following hypotheses were proposed, suggesting that pseudocirrhosis develops due to: (1) a response to chemotherapy by metastatic liver tissue resulting in scarring and capsular retraction, (2) fibrosis combined with infiltrating hepatic metastatic masses, (3) a nodular regenerative hyperplasia in response to ischemia from a chemotherapy-induced hepatic injury, and (4) sinusoidal occlusion caused by chemotherapy-related injury [1,5-8]. Recent reports suggest that chemotherapy-induced liver injury may be critical to the pathogenesis of pseudocirrhosis, because approximately all patients had previously received systemic chemotherapy [3,9]. In our case, the liver biopsy showed cancer cell infiltration consistent with the fibrotic area which was similar to the fibrosis seen in liver cirrhosis, and no fibrosis was observed in the remaining normal hepatic tissue. This characteristic form of hepatic infiltration and fibrosis may be related to the response of the metastatic liver tumors to chemotherapy. Many of the previously reported cases of pseudocirrhosis, in which the liver pathology could be evaluated, showed intrahepatic tumor infiltration with fibrosis, as in our case, suggesting that these characteristic tumor forms may be associated with portal hypertension in pseudocirrhosis [5,7,10-12].

Liver biopsy is useful in diagnosing diffuse tumor infiltration of the liver in patients with pseudocirrhosis. Pathologically, pseudocirrhosis almost always shows diffuse tumor invasion in the liver, and imaging studies do not always reveal focal lesions suggestive of liver metastases [10]. In the present case, no findings suggestive of intrahepatic metastatic lesions were revealed on any imaging examination, such as enhanced CT, Gd-EOB-DTPA, or 18F-FDG PET-CT. Therefore, a liver biopsy may be considered for the diagnosis of diffuse liver metastases, even if the images show no evidence of focal liver lesions.

However, there is a need for serum biomarkers that can effectively provide an early diagnosis and replace the existing invasive liver biopsy. Although pseudocirrhosis is a rapidly progressive and clinically important condition, early diagnosis is difficult because it is asymptomatic before progression like many other liver diseases. Moreover, biomarkers for the early diagnosis of pseudocirrhosis have not been reported. In our case, Mac-2 binding protein glycosylation isomer, hyaluronic acid, type-4 collagen 7S, and the FIB-4 index were all high at the time of pseudocirrhosis diagnosis. All of these have been established as useful biomarkers for the early detection of liver fibrosis [13]. Therefore, they may be useful for the early diagnosis of pseudocirrhosis as well. In particular, the FIB-4 index increased over time with the progression of pseudocirrhosis. However, it is difficult to evaluate the FIB-4 index alone as a biomarker for pseudocirrhosis, because the FIB-4 index can be easily altered by chemotherapy-induced liver injury and thrombocytopenia (Figure 2). Thus, more studies are required in order to evaluate and correlate these markers to pseudocirrhosis.

## Conclusions

Pseudocirrhosis is a life-threatening condition that often develops in breast cancer chemotherapy patients with liver metastases. Since the cause may be a desmoplastic response of metastatic liver cancer to chemotherapy, biopsy may need to be considered even if there is no obvious liver tumor on imaging. Pseudocirrhosis should be considered as a possible severe complication in patients with advanced breast cancer. Further accumulation of cases and further studies are needed to clarify the biomarkers for the early diagnosis of pseudocirrhosis.
